# Spontaneous Pneumomediastinum Revealing Asthma: The Macklin Effect

**DOI:** 10.7759/cureus.24978

**Published:** 2022-05-13

**Authors:** Chaynez Rachid, lina Romane, Salma Ait Batahar, Lamyae Amro

**Affiliations:** 1 Department of Pneumology, Ar-Razi Hospital, Centre Hospitalo-Universitaire (CHU) Mohammed VI, Marrakech, MAR; 2 Department of Pulmonology, Ar-Razi Hospital, Centre Hospitalo-Universitaire (CHU) Mohammed VI, Marrakech, MAR

**Keywords:** macklin effect, sub cutaneous emphysema, dyspnea, asthmatic, pneumomediastinum

## Abstract

Pneumomediastinum is defined by the presence of air in the mediastinum, which may be either secondary to trauma, pneumothorax or perforation of the airways, or spontaneous. We report the case of a 28-year-old female patient with pneumomediastinum revealing asthma in acute exacerbation. The patient wasn’t known to be asthmatic or to have an atopic background, no history of surgery, nor any notion of trauma, or recent iatrogeny. She presented with sudden onset of tachypnea associated with chest tightness and productive cough with greenish sputum. Auscultation of her chest revealed audible sibilant rales with the presence of subcutaneous emphysema. Chest radiograph objectivated an aeric border along the edge of the cardiac silhouette associated with subcutaneous hyperclarity of the cervical region. The thoracic CT scan confirmed the presence of a diffuse moderate pneumomediastinum. The patient was put under nasal oxygen, nebulized Ventolin and given intravenous corticosteroid therapy. The patient evolved favorably within three days marked by clinical improvement, the persistence of discrete sibilant rales at the apexes, as well as subcutaneous emphysema in regression after oxygen therapy and conventional medical treatment.

## Introduction

Spontaneous pneumomediastinum is defined by the presence of air in the mediastinum, which may be secondary to trauma, pneumothorax or perforation of the airways, or spontaneous. The latter occurs outside of any traumatic, iatrogenic context but can be caused by underlying lung disease, in particular decompensated asthma.

It is a rare condition, clinically suspected in the presence of retrosternal pain, or subcutaneous emphysema which is highly suggestive but not pathognomonic, either simply presents as the presence of clinical signs that define an asthma attack. This complication, however, can be life-threatening if not recognized early and properly managed. The risk of tension pneumomediastinum (PM) justifies close clinical follow-up in the intensive care unit (ICU), because its potential complications may demand a more aggressive approach. The diagnosis is subsequently confirmed by a chest x-ray coupled with a chest CT scan which will show the presence of air in the mediastinum and subcutaneously. Here, we report a case of spontaneous pneumomediastinum in an asthmatic patient.

## Case presentation

**Figure 1:**
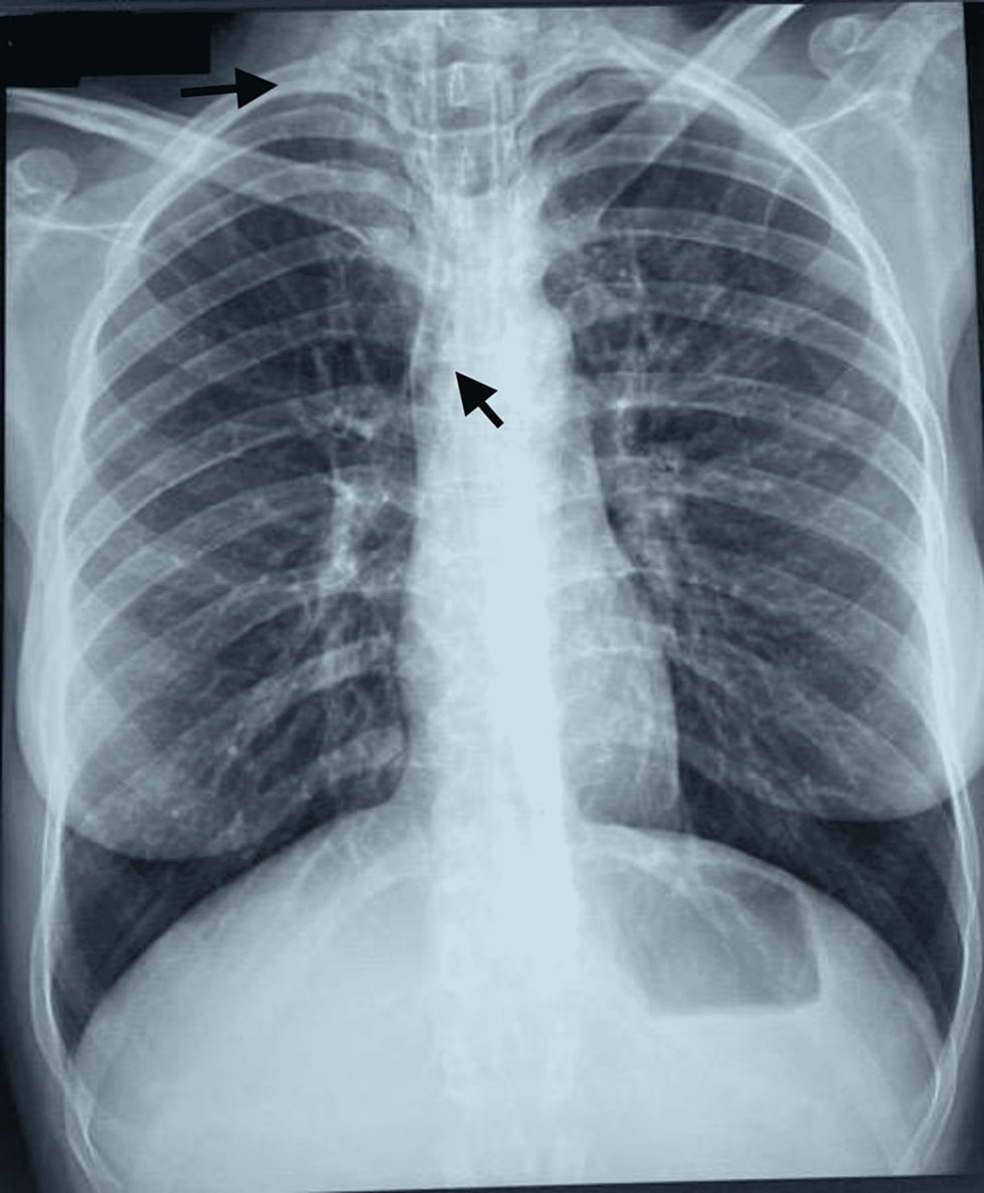
Posteroanterior chest radiograph demonstrates typical features of pneumomediastinum. The image shows aerial border along the edge of the cardiac silhouette associated with subcutaneous hyperclarities of the cervical region, drop heart appearance, and thoracic distension.

The patient was a 28-year-old housewife not known to be asthmatic or to have an atopic background, with no known drug allergy, no history of diabetes, nor any notion of trauma or recent iatrogeny. She consulted the emergency room for wheezing dyspnea with chest tightness. The history of the disease goes back to two days, with the sudden onset of polypnea associated with chest tightness, and productive cough with greenish sputum. The clinical examination found a polypneic patient with 32 cycles per minute with suprasternal and subcostal draught, heart rate of 103 beats per minute. Pulmonary auscultation finds audible sibilant rales in both pulmonary hemifields, as well as basicervical and anterosuperior thoracic crepitations signaling the presence of subcutaneous emphysema. Oxygen saturation was 93% on room air, absence of cyanosis with normal speech. Chest radiograph showed an aeric border along the edge of the cardiac silhouette associated with subcutaneous hyperclarity of the cervical region, thoracic distension, and drop heart appearance (Figure 1).

**Figure 2:**
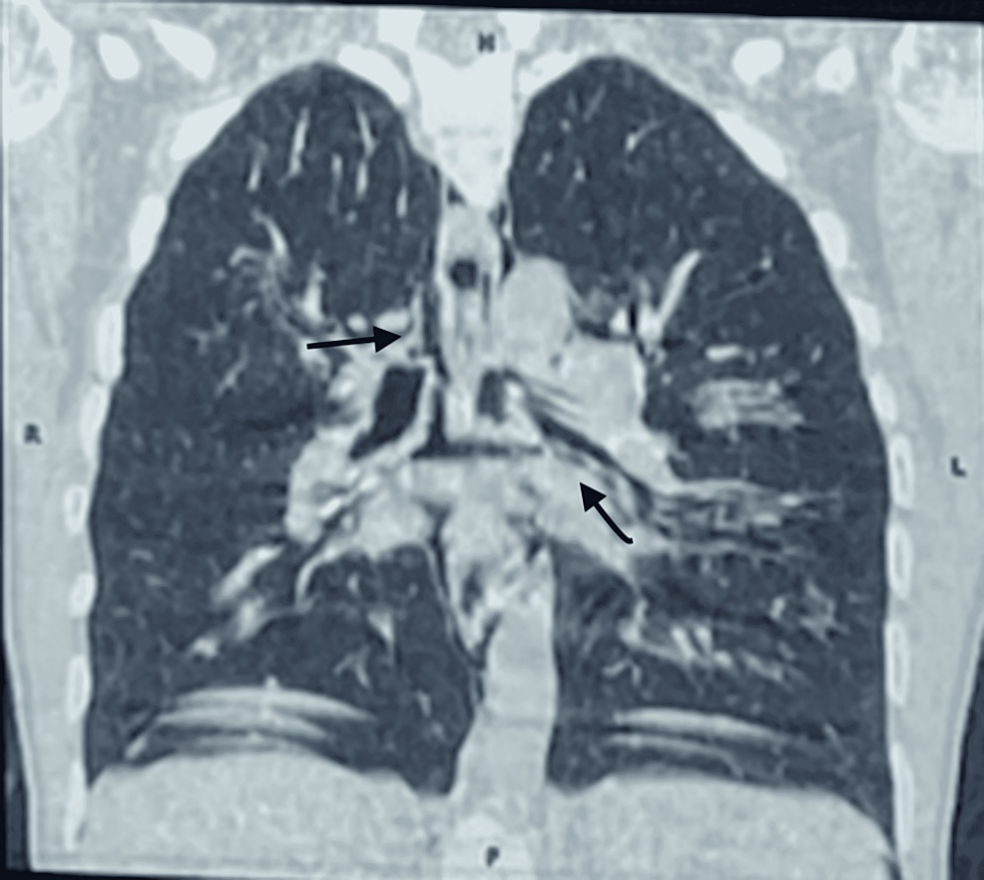
Thoracic CT scan demonstrates a massive pneumomediastinum. The CT scan image confirms air collection along the perivascular connective tissue, the Macklin effect in the peripheric area and the perihilar area, and massive pneumomediastinum.

**Figure 3:**
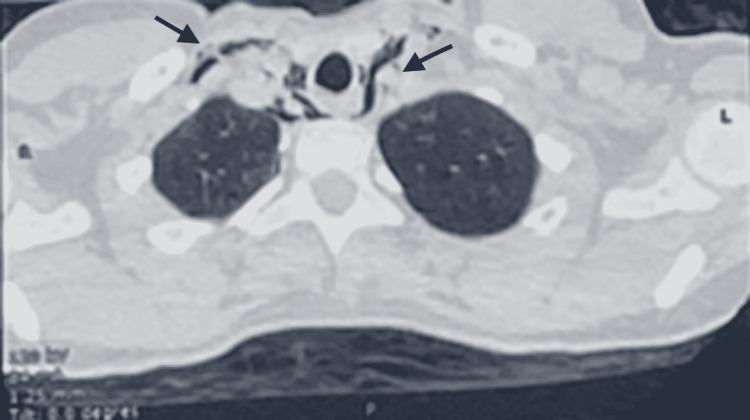
Thoracic CT scan images showing pneumomediastinum and cervical subcutaneous emphysema.

**Figure 4:**
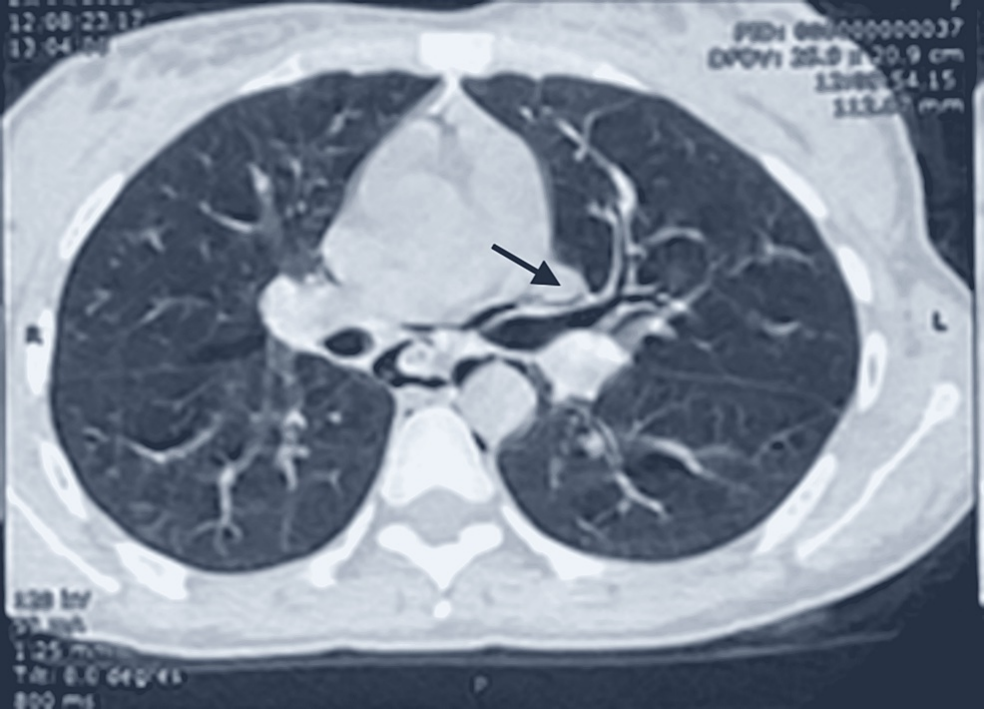
Thoracic CT scan showing the Macklin effect. The CT scan image confirms the Macklin effect by demonstrating air dissection along the peribronchovascular sheaths.

The thoracic CT scan showed the presence of a diffuse moderate pneumomediastinum, with extensive subcutaneous cervical emphysema and a small subpleural retractive parenchymal lesion with a small calcified nodule in the ventral segment of the right lower lobe and Air dissection along the peribronchovascular sheaths (Figures 2-4).

The patient was put under nasal oxygen glasses at 4 L/min, nebulized Ventolin/Atrovent every 20 min during the first hour and then every one hour then every four hours until improvement, and intravenous corticosteroid therapy at a rate of 80 mg of methylprednisolone. The evolution was marked by clinical improvement, the persistence of discrete sibilant rales at the apexes, as well as subcutaneous emphysema in regression.

## Discussion

Spontaneous pneumomediastinum is defined as the presence of air in the mediastinal structures without any obvious cause. It is a rare clinical entity that most commonly affects young adults. Spontaneous pneumomediastinum was first reported in 1939 by Hamman [1,2].

The pathophysiological mechanisms of spontaneous pneumomediastinum are poorly defined and the hypothesis most often reported in the literature is that of an endobronchial hyper pressure with closed glottis. This hyper pressure would be responsible for alveolar rupture near the vascular septa, the latter draining the aerated effusion thus created towards the mediastinum. This is a common mechanism with some spontaneous pneumothoraxes [3]. There does not seem to be any really identified favoring factor, although some series report a number of asthmatic patients for whom it may be a mode of revelation of the disease [4].

The case described here shows the alveolar rupture with air dissection along the interstitial sheaths toward the mediastinum, eventually leading to a pneumomediastinum. Alveolar rupture is responsible for more than 95% of cases of pneumomediastinum and is more frequent than esophageal or tracheobronchial disruptions [5]. It is called the Macklin effect and it is identified on chest CT scan by visualizing air dissection along perivascular and peribronchial sheaths, but CT scan confirmation is unnecessary [6]. The Macklin effect has also been implicated in 39% of cases of pneumomediastinum following severe blunt chest trauma [7].

In our observation, we note the exacerbation of the asthma attack and the effort of coughing, which were incriminated in the occurrence of pneumomediastinum in our patient. The typical clinical form is that of a picture of retrosternal chest pain occurring in a young adult. In the literature, this functional sign is present two out of three times. A persistent cough is observed in 50% of patients and cervical discomfort in a third of them. Dyspnea and dysphagia are rarer and should call into question the spontaneity of pneumomediastinum. The examination sign, key to the diagnosis, is subcutaneous emphysema usually localized to the left hemithorax and the anterior cervical region. Hamman's sign is an auscultatory crepitation synchronous with the heartbeat, pathognomonic of pneumomediastinum [8]. The positive diagnosis is made by a frontal thalamic radiograph which shows an aerial border along the left edge of the cardiac silhouette and subcutaneous hyperclarities in the cervical region [9].

The natural evolution of spontaneous pneumomediastinum is towards recovery in 48-96 hours with complete disappearance of clinical and radiological signs. No specific therapy, in particular antibiotic and oxygen therapy, seems to have proven its effectiveness. Recurrences appear to be rare. Complications are exceptional, however, cases of compressive pneumomediastinum with tamponade requiring surgical drainage have been described [10].

Spontaneous pneumomediastinum is therefore the only clinical event in which the presence of air in the media should not be considered serious and should not trigger a multitude of additional examinations on a routine basis. In cases of lesions of the tracheobronchial tree or especially the esophagus, the initial clinical picture may be paucisymptomatic, the risk of evolution towards mediastinitis is major. In this context, the presence of fever, dysphagia, or dyspnea is the warning sign of secondary pneumomediastinum. A cervicothoracic CT scan and an endoscopic assessment of the aerodigestive tract should then be performed rapidly. If the CT scan with injection and ingestion of contrast medium allows most of the time to demonstrate the esophageal lesions by indirect signs, the endoscopic assessment remains essential to better define the situation of a possible esophageal lesion and the existence of underlying esophageal pathology [11]. These last two pieces of information are crucial for the choice of the approach and the decision of conservative treatment in case of esophageal rupture which is always managed surgically [12]. Nevertheless, as demonstrated by this presented clinical case, the history of the disease and the clinic allow a clear identification of spontaneous pneumomediastinum provided that the patient is familiar with this pathology.

## Conclusions

Spontaneous pneumomediastinum is a very rare and benign entity and is defined by the presence of free air within the mediastinum. A favorable circumstance is found, through an increase in alveolar pressure at the origin of the Macklin effect. Primary spontaneous pneumomediastinum (PMSp) is a rare pathology of the young subject and in the vast majority of cases benign. Although it is generally easy to distinguish between PMSp and secondary pneumomediastinum, the elements that may point to an esophageal rupture must be known. Treatment of PMSp should be conservative and based on pain relief. There is no point in instituting systematic antibiotic prophylaxis. A hospitalization of 24-48 hours seems reasonable.

## References

[1] Ryoo JY (2012). Clinical analysis of spontaneous pneumomediastinum. Tuberc Respir Dis (Seoul).

[2] Sahni S, Verma S, Grullon J, Esquire A, Patel P, Talwar A (2013). Spontaneous pneumomediastinum: time for consensus. N Am J Med Sci.

[3] Langwieler TE, Steffani KD, Bogoevski DP, Mann O, Izbicki JR (2004). Spontaneous pneumomediastinum. Ann Thorac Surg.

[4] Shennib HF, Barkun AN, Matouk E, Blundell PE (1988). Surgical decompression of a tension pneumomediastinum. A ventilatory complication of status asthmaticus. Chest.

[5] Fernandes D, Pereira S, Guedes C, Silva D (2020). Massive traumatic subcutaneous emphysema. Acta Med (Hradec Kral).

[6] di Castelguidone ED, Pinto A, Merola S, Stavolo C, Romano L (2005). Role of spiral and multislice computed tomography in the evaluation of traumatic and spontaneous oesophageal perforation. Our experience. Radiol Med.

[7] Murayama S, Gibo S (2014). Spontaneous pneumomediastinum and Macklin effect: overview and appearance on computed tomography. World J Radiol.

[8] Sakai M, Murayama S, Gibo M, Akamine T, Nagata O (2006). Frequent cause of the Macklin effect in spontaneous pneumomediastinum: demonstration by multidetector-row computed tomography. J Comput Assist Tomogr.

[9] Okada M, Adachi H, Shibuya Y, Ishikawa S, Hamabe Y (2014). Diagnosis and treatment of patients with spontaneous pneumomediastinum. Respir Investig.

[10] Panacek EA, Singer AJ, Sherman BW, Prescott A, Rutherford W (1992). Spontaneous pneumomediastinum: clinical and natural history. Ann Emerg Med.

[11] Wintermark M, Schnyder P (2001). The Macklin effect: a frequent etiology for pneumomediastinum in severe blunt chest trauma. Chest.

[12] Jougon J, Mc Bride T, Delcambre F, Minniti A, Velly JF (2004). Primary esophageal repair for Boerhaave's syndrome whatever the free interval between perforation and treatment. Eur J Cardiothorac Surg.

